# Sum-Frequency Generation Spectroscopy of Plasmonic Nanomaterials: A Review

**DOI:** 10.3390/ma12050836

**Published:** 2019-03-12

**Authors:** Christophe Humbert, Thomas Noblet, Laetitia Dalstein, Bertrand Busson, Grégory Barbillon

**Affiliations:** 1 Univ Paris-Sud, Université Paris-Saclay, Laboratoire de Chimie Physique, CNRS, Batiment 201 P2, 91405 Orsay, France; thomas.noblet@u-psud.fr (T.N.); dalstein@gate.sinica.edu.tw (L.D.); bertrand.busson@u-psud.fr (B.B.); 2 EPF-Ecole d’Ingénieurs, 3 bis rue Lakanal, 92330 Sceaux, France

**Keywords:** nanoparticles, non-linear optics, surface plasmons, sum-frequency generation spectroscopy, interfaces, gold

## Abstract

We report on the recent scientific research contribution of non-linear optics based on Sum-Frequency Generation (SFG) spectroscopy as a surface probe of the plasmonic properties of materials. In this review, we present a general introduction to the fundamentals of SFG spectroscopy, a well-established optical surface probe used in various domains of physical chemistry, when applied to plasmonic materials. The interest of using SFG spectroscopy as a complementary tool to surface-enhanced Raman spectroscopy in order to probe the surface chemistry of metallic nanoparticles is illustrated by taking advantage of the optical amplification induced by the coupling to the localized surface plasmon resonance. A short review of the first developments of SFG applications in nanomaterials is presented to span the previous emergent literature on the subject. Afterwards, the emphasis is put on the recent developments and applications of the technique over the five last years in order to illustrate that SFG spectroscopy coupled to plasmonic nanomaterials is now mature enough to be considered a promising research field of non-linear plasmonics.

## 1. Introduction

Presently, the use of the physico-chemical properties of nanomaterials [1,2,3] in everyday life has become a reality, whether in electronic instruments or medical applications. Thus, their antiseptic action is, for instance, used in razor blades covered with a coating of silver nanoparticles (AgNPs). They are also introduced in some medical treatments such as in cancer therapy to selectively destroy some tumors. Besides, the society developed an interest for a better understanding of the particle interaction with their environment. More generally, metallic nanoparticles arouse a growing interest by the combination of their unique properties with promising developments in areas as diverse as catalysis [4], biophysics [5] or medicine [6], especially by taking advantage of their optical properties in the visible spectral range as in the case of gold nanoparticles (AuNPs) [7,8,9,10]. More technologically advanced applications also appeared, in computer processors for instance, and some of these nanoparticles are incorporated in medical treatments such as cancer therapy wherein they are employed to selectively kill tumors [11]. Nevertheless, at this nanoscale, monitoring the action of nano-objects on their immediate environment at molecular level is a sensitive issue because many of their above-mentioned properties are not well understood, which may pose risks to the living body. To better understand these issues of toxicity and health potential for the society, new research tools adapted to the study of interactions between nano-objects and their environment are absolutely necessary [3]. Among the numerous techniques studying nanoparticles such as X-ray spectroscopies [12] or scanning probe microscopies (Atomic Force Microscopy, Scanning Tunneling Microscopy) [13], non-linear optics based on Sum-Frequency Generation (SFG) spectroscopy [14] is distinguished by its surface specificity which allows addressing various fundamental or applied issues related to the chemical functionalization of materials (metals, semiconductors, insulators) from the microscale to the atomic scale, over both the infrared (IR) and visible spectral ranges. The latter affords complementary information with respect to PM-IRRAS (Polarization Modulation-Infrared Reflection-Adsorption Spectroscopy) [15] and SERS (Surface-Enhanced Raman Spectroscopy) [16,17,18,19]. Moreover, SFG is now a competitive tool: its sensitivity has indeed increased over the past few years thanks to technical and scientific developments at the picosecond and femtosecond time scales. The kinetics and dynamics of the vibrational electronic coupling, charge and energy transfers constitute the core of the physico-chemical properties that can be investigated in this way [20,21,22,23,24,25].

At the nanoscale, SFG was first performed in the 1990s on nanomaterials such as Pd and Pt nanoparticles used as catalysts in a gaseous environment in order to reach higher conversion efficiency of carbon monoxide (CO) in carbon dioxide (CO_2_) as a function of pressure or/and temperature with respect to their crystalline counterpart [26,27]. It also includes some very recent work concerning Li-Ion batteries based on silicon nanoparticles [28]. From the beginning, SFG was also used to unveil the adsorption/desorption mechanisms on the catalysts surface as reported in numerous studies [29,30]. The first experiment on nanoparticles at the air/liquid interface was reported in 2000 on a cationic surfactant deposited on AuNPs stabilized in their anionic form and forming a monolayer (not very stable) on a silicon substrate [31]. The presence of the surfactant on the AuNPs was thus demonstrated as well as a potential effect of amplification of the surfactant signal. From this moment, the hypothesis of an amplification of the non-linear optical response of the material by the simultaneous excitation of the AuNP surface plasmon resonance (SPR) has been assumed. Indeed, the classic SFG process is produced by mixing two incident IR and visible laser beams on the sample as developed and explained in the corresponding section. With the visible laser beam, it is likely to probe electronic transitions: metal interband transitions, charge transfers, molecular electronic transitions, and SPRs of nanoparticles.

## 2. Sum-Frequency Generation Spectroscopy of Metallic Nanoparticles

### 2.1. Fundamentals of Sum-Frequency Generation Spectroscopy

**Figure 1:**
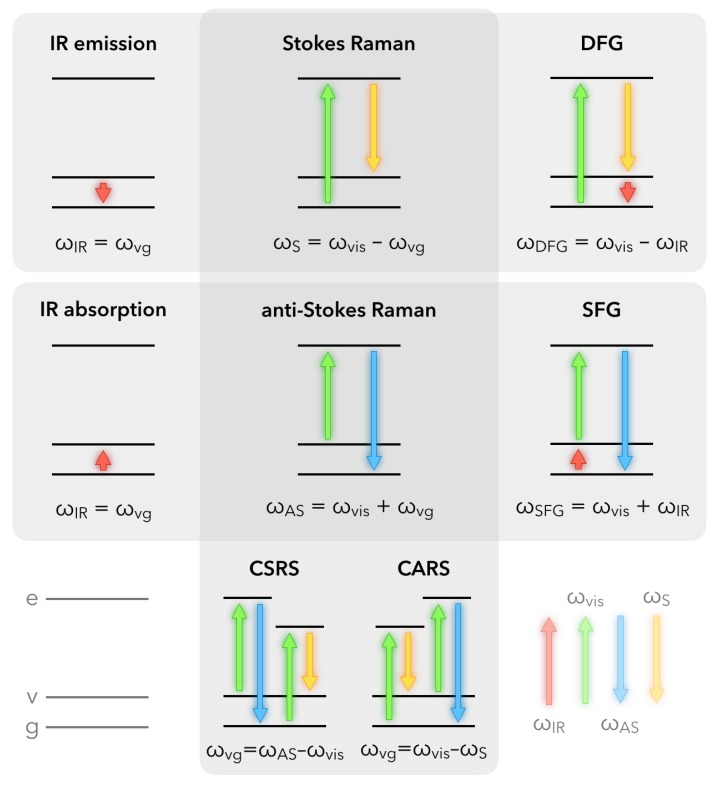
Molecular energy diagrams used for IR, Raman, SFG/DFG and CARS/CSRS spectroscopy. SFG = Sum-Frequency Generation; DFG = Difference-Frequency Generation; CARS = Coherent Anti-Stokes Raman Scattering; CSRS = Coherent Stokes Raman Scattering; S = Stokes; AS = Anti-Stokes; Energy level code: e = virtual electronic state, v = vibrational state, g = ground state.

For molecules, optical processes can be described in terms of energy diagrams. In a practical way, with respect to the surface or bulk properties of materials, non-linear optical spectroscopies up to the order 3 are routinely used, for example through the 4-photon mixing encountered in Coherent Anti-Stokes Raman Scattering (CARS) or Coherent Stokes Raman Scattering (CSRS). The reader interested in a comparison of CARS developments on metallic nanostructures with respect to some SFG results can refer to the review written in 2014 by Lis and co-workers [32]. It is worth noting that in a general way, first order spectroscopies such as IR/UV-Visible absorption and emission allow probing of the linear first order susceptibility $\chi^{\left(1\right)}$ of materials, while Second Harmonic Generation (SHG) and Sum/Difference-Frequency Generation (SFG/DFG) spectroscopies give access to the non-linear optical second-order susceptibility $\chi^{\left(2\right)}$. To finish, Raman/SERS/CARS spectroscopies probe the non-linear third order susceptibility $\chi^{\left(3\right)}$. Figure 1 summarizes in terms of simple molecular energy scheme how non-linear processes of order 2 and 3 occur. It also emphasizes the differences and links that exist between them and the basic techniques of infrared and Raman spectroscopies. The common point of these techniques is obviously the access to specific vibration modes $\omega_{\mathrm{vg}}$ of the constitutive molecules present in the studied materials. It is worth noting that the Raman and non-linear processes exist even in the absence of real electronic states in the molecule. Conversely, if the visible laser beam is tuned to match the energy of real electronic states, we may significantly enhance the efficiency of these mechanisms and improve the sensitivity detection threshold, as performed in Resonant Raman or Two-Color Sum-Frequency Generation (2C-SFG) spectroscopies. In the latter case, it becomes possible to highlight vibronic coupling inside molecules, which is called Doubly Resonant Sum-Frequency Generation (DRSFG) process [33]. When molecules are adsorbed on a (nanostructured) interface, 2C-SFG can benefit from the electronic properties of the substrate to amplify, under certain conditions, the molecular response through coupling mechanisms between both constituents: molecular adsorbate and substrate [34,35,36,37,38].

**Figure 2:**
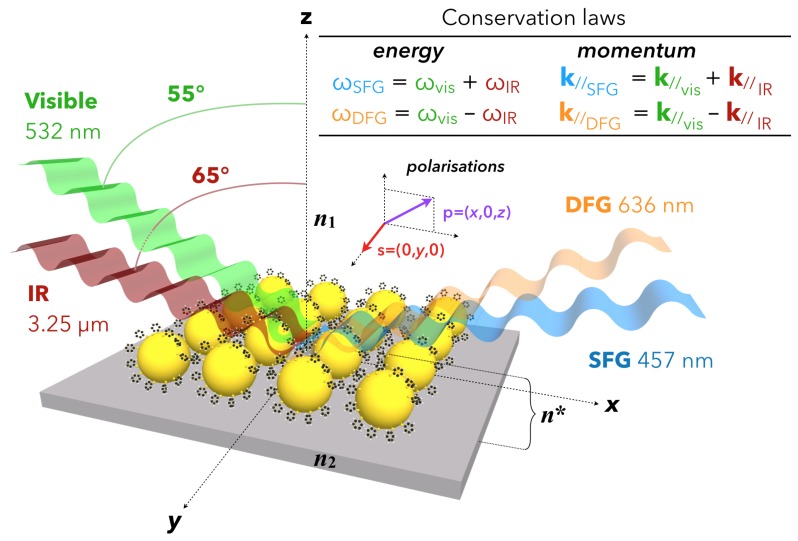
Sketch of SFG/DFG spectroscopy at a nanostructured interface: Si/AuNPs + adsorbed thiophenol/ambient air. The refractive index of each medium constituting the interface is noted $n_{i}$ ($n_{1}$ for air, $n_{2}$ for silicon) while that of the interface, noted n*, must be calculated. The common polarization plane used in SFG spectroscopy depicts the geometry of the linear polarization *p* (*x*,0,*z*) and *s* (0,*y*,0) of the three laser beams depending of the chosen experimental scheme.

Fundamentally, SFG spectroscopy is based on a non-linear second-order optical process involving three photons mixed in a medium or at an interface, as illustrated in Figure 2 in the case of functionalized nanoparticles grafted on silicon. To be efficient, it requires intense laser beams to interact coherently at the same point of the probed interface so that the energy ($\omega_{\mathrm{SFG}}=\omega_{\mathrm{VIS}}+\omega_{\mathrm{IR}}$) and the parallel component (*x*-direction) of the momentum ($k_{\mathrm{SFG}}=k_{\mathrm{VIS}}+k_{\mathrm{IR}}$) conservation rules are met. As drawn in this picture, similar relationships are obtained for Difference-Frequency Generation (DFG) spectroscopy. The interest of this complementary sister spectroscopy will be evidenced later for metallic nanoparticles.

Due to the complex structure of such an interface, each of the three interfacial components (related to their respective refractive indices $n_{1}$ for air, $n_{2}$ for silicon and n* for the AuNPs/thiophenol) drawn in Figure 2 has to be considered in the description of the SFG process. The access to these quantities depends on the choice of the electric field polarization of the IR, Visible, and SFG beams. In our case, as depicted in this figure and following the convention of the literature for the SFG processes, the three beams have always a linear polarization defined either parallel (*p*) or perpendicular (*s*) to the incidence plane (xz) of the three involved beams. In other words, *p*-polarization refers to *x*- and *z*-components of the electric fields while *s*-polarization refers to the *y*-component of the electric fields. In the framework of the electric dipole approximation, and considering the molecular energy levels depicted in Figure 1, the non-linear SFG/DFG processes simultaneously follow the IR and Raman molecular selection rules. In other words, a vibrational transition is detected in SFG spectroscopy if it is both IR and Raman-active, which generally happens upon adsorption on any substrate. Indeed, the adsorption process soften the spectroscopic exclusion rules between IR and Raman processes, allowing probing of molecules of simple or complex geometry such as carbon monoxide (CO) or Buckminsterfullerenes (C_60_) in chemical interaction with platinum or silver, respectively. As an interface constitutes the place where the centrosymmetry of the electronic properties in the bulk of two different media is broken, SFG spectroscopy is a choice technique to probe complex surfaces and interfaces because it remains intrinsically blind with respect to the bulk contributions located on both sides of the target area (e.g., molecules in the gas phase or in solution). On the contrary, IR and Raman spectroscopies encompass bulk and interfaces contributions, at the exception of PM-IRRAS and SERS for particular metallic substrates.

**Figure 3:**
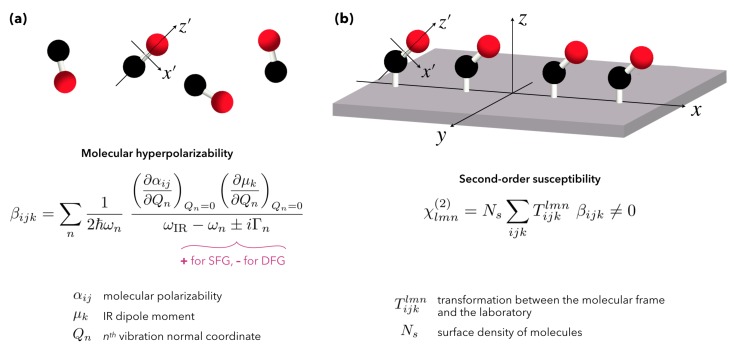
Microscopic view of the hyperpolarizability β of a carbon monoxide molecule (**a**) and macroscopic view of the non-linear second-order susceptibility $\chi^{\left(2\right)}$ of a carbon monoxide layer adsorbed on a platinum substrate (**b**). β and $\chi^{\left(2\right)}$ are third-rank tensors with 27 components that can be probed by SFG spectroscopy as a function of the SFG, Visible and IR laser beams polarization: *p* or *s* as defined in Figure 2. These non-linear physical values are related through a coordinate transformation *T* from the molecular frame to the laboratory (interface).

The second-order polarizability (hyperpolarizability: β) characterizing molecules in SFG is directly related to a change in the Raman polarizability α and the IR dipole moment μ of the vibrational transitions involved in the process. Moreover, we notice a direct link between β (molecule) and $\chi^{\left(2\right)}$ (macroscopic) via a coordinate transformation *T* making it possible to switch from the molecule reference system to that of the whole sample as illustrated in Figure 3 for the carbon monoxide. Actually, the symmetry rules in the 3-photon mixing of SFG/DFG mean that the quantities β and $\chi^{\left(2\right)}$ are third-rank tensors, i.e., they have 27 components measurable by multiple combinations of the possible orientations (polarizations) of the 3 associated electric fields (SFG, Visible, Infrared) in each direction (*x*, *y*, *z*) of our three-dimensional space. Experimentally, we usually measure $\chi^{\left(2\right)}$ and we can theoretically retrieve β, taking into account the average over all molecular orientations in the macroscopic interface. Given the complexity of the β formulation illustrated very early by Franken [39], Ward [40] and later by Hirose [41] for transformation *T*, this is currently done only for simple cases with well-defined geometries and controlled geometries, and when only the molecular signal contributes to the SFG response for instance in catalysts [42]. Nevertheless, this is already partially done for basic structures in order to find the average orientation of molecules deposited on a surface in the case of Self-Assembled Monolayers (SAM) or polymers, and even for biosurfaces [43]. In summary, at least one component of the $\chi^{\left(2\right)}$ of any interface should be non-zero in order to detect SFG signal. The researcher interested by a comprehensive and pedagogic description of the microscopic and macroscopic relations between β and $\chi^{\left(2\right)}$ on any kind of interface can read the tutorial approach of SFG spectroscopy elaborated by Lambert et al. in reference [44]. Hence provided with the basics of molecular SFG spectroscopy, we can now extend it to nanostructured interfaces in order to highlight the role played by the optical properties of nanoparticles.

In Figure 2, we have to take into account all the components of the interface including those of the substrate, by giving a complete description of the effective refractive index n*. Indeed, for SFG spectroscopy performed at interfaces, the substrate cannot be neglected: for instance, in the case of metal or semiconductor materials, wherein $\chi^{\left(2\right)}$ is non-zero, the substrate often gives rise to a more intense SFG signal than the molecules because of the free and/or bound electrons present in the materials. The strong illumination by the incident visible laser beam of these objects translates into the excitation of the electron interband and intraband electronic transitions in metals or the creation of excitons (electron-hole pairs) in semiconductors. In both cases, the substrate generates the so-called non-resonant SFG contribution. At the opposite, the molecular SFG signal is called resonant (with respect to the IR energy) when the incident IR laser beam frequency corresponds to that of the molecular vibration modes. For the sake of clarity, the case of molecules exhibiting electronic transitions in the visible spectral range will not be considered in this report. The interested reader will nevertheless find experimental results showing some adsorbed molecules which are resonant in both IR and visible spectral ranges, evidencing vibro-electronic SFG processes in various kinds of physical [45,46,47], chemical [33] and biological [48] systems. The description of the SFG processes arising from gold surfaces is a difficult task that has been extensively addressed experimentally and theoretically because it must take into account the non-linear contribution coming from the surface and the bulk of the specific electronic properties of this metal in the visible spectral range. A clear and complete description of the physics at play in its $\chi^{\left(2\right)}$ is reported in reference works [49,50,51], based on SFG measurements performed on molecular monolayers adsorbed on flat gold surfaces. In these works, the molecules act as a reference probe of the electronic properties of gold because the SFG spectral shape of the vibration modes (dips, peak, Fano) is modulated by the color choice of the incident visible laser beam ranging from violet to red. This is based on a three-layer model similar to the image depicted in Figure 2 by considering air/molecules/gold instead of air/AuNPs+thiophenol/Si. It has been shown that the contribution of the free and bound electrons explains the shape modulation of the SFG spectra at this interface [52]. To date, such models have again to be applied to the nanostructured interfaces depicted in Figure 2. Moreover, in the SFG process, the incident visible wavelength is set to 532 nm in order to enhance the AuNPs localized surface plasmon resonance (LSPR) located between 520 and 540 nm corresponding to the case of gold spheres with a diameter of 15 nm. The IR wavelength set at 3.25 µm in the SFG process is chosen to be resonant with the C–H stretching vibration mode of the phenyl ring. This is not related to LSPR properties but to the molecular vibrations of the thiophenol adsorbed on AuNPs. It allows demonstration of the LSPR-SFG coupling, i.e., the enhancement of the C-H vibration mode in the LSPR electric field. In the case of AuNPs, choosing a 632 nm or 785 nm visible wavelength will not allow easy demonstration of a LSPR-SFG coupling because it is located too far from the LSPR maximum at 532 nm (see Figure 1 and Section 2.2).

### 2.2. Nanoparticles and Sum-Frequency Generation: The Gold Case

**Figure 4:**
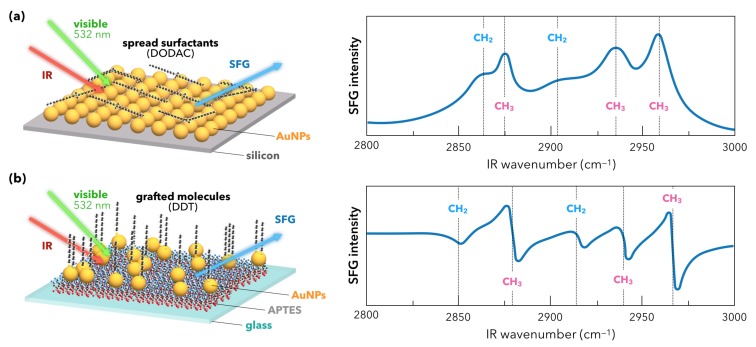
Sketch of the first reference SFG experiments performed on interfaces constituted of 15 nm diameter AuNPs synthesized by Turkevich method. (**a**) Scheme of the DODAC/AuNPs/Si interface (no grafting of AuNPs or DODAC molecules at any step). Each constituent is just deposited or spread, respectively. The corresponding SFG signal displays the five strong methyl and methylene peak-shaped vibration modes of the DODAC molecules (picture adapted from reference [31]). (**b**) Scheme of the DDT/AuNPs/Glass interface (grafting of AuNPs on glass through APTES and chemical functionalization of AuNPs by DDT molecules). The corresponding SFG signal displays the five weak methyl and methylene dip-shaped vibration modes of the DDT molecules (picture adapted from reference [53]).

**Figure 5:**
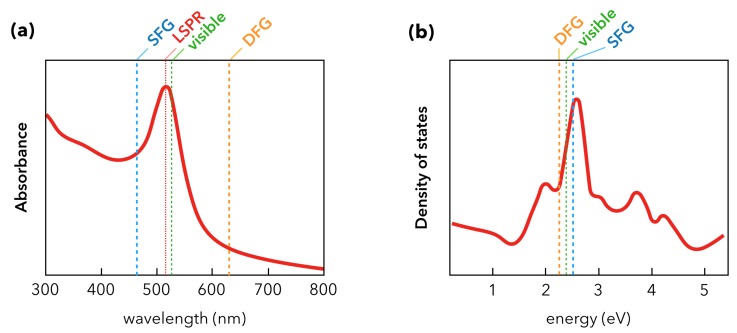
Electronic and optical properties of gold. (**a**) Localized surface plasmon resonance (LSPR) of AuNPs in aqueous solution displayed as a function of the incident wavelength as measured by UV-Visible spectroscopy. (**b**) Scheme of the density of electronic states (DOS) of bulk gold as a function of the incident energy. Pictures (**a**,**b**) are tagged with the SFG and DFG energy for an incident visible beam of 532 nm wavelength and an incident IR beam of 3 µm. In these conditions, it is clear that although the SFG/DFG/visible beams are located near the LSPR maximum in (**a**), the contribution of the gold DOS (main s−d interband electronic transition located at 487 nm) in (**b**) may interfere and drastically hamper a possible LSPR-SFG coupling.

The founding SFG spectroscopic measurements on AuNPs were performed as illustrated in Figure 4 by the Davies [31] and Tadjeddine [53] teams in 2000 and 2005, respectively. The nanoparticles were synthesized by the classical Turkevich method [54]. In these conditions, they have a spherical shape of 15 nm diameter and are spread or grafted on silicon or glass substrate, respectively. As for the latter, AuNPs are grafted by silanization of the glass substrate with a monolayer of APTES molecules (3-(aminopropyl)triethoxysilane). The incident visible laser beam is fixed in the green (532 nm) while the incident IR beam is tuned between 2800 cm^−1^ and 3000 cm^−1^. The molecular probe of the AuNPs surface in Figure 4a is a surfactant co-adsorbed with the AuNPs (dioctadecyldimethylammonium chloride, DODAC) while it is chemically grafted after deposition of the AuNPs in Figure 4b (dodecanethiol, DDT). In both cases, five typical vibration modes are detected by SFG spectroscopy. They come from the DODAC and DDT molecules and are located at IR wavenumbers corresponding to the vibrational fingerprint of the methyl (CH_3_) and methylene (CH_2_) vibration modes as pointed on both SFG spectra. Nevertheless, we observe that their spectral shapes differ. This effect is characteristic of SFG spectroscopy and, as highlighted in the previous section, finds its origin in the non-resonant contribution of the interface. In other words, the different ways the DODAC and DDT molecules interact with the AuNPs and the silicon/glass substrates after the surface preparation is put in evidence by SFG measurements. In Figure 4, vibration modes appear peak- or dip-shaped. In the case of DODAC on AuNPs/Si, the strong reflectivity of the interface and the high surface density of AuNPs related to the surface preparation favors molecular detection in SFG. The latter is amplified by the LSPR coupled to the vibration modes because of the enhancement of the surrounding electric field. The LSPR of a gold colloidal solution is evidenced by UV-Visible absorption spectroscopy as depicted in Figure 5a.

This enhancement is not clear for DDT adsorbed on AuNPs/glass given the poor reflectivity of glass with respect to silicon and the weaker AuNPs surface density. The broad and strong gold interband s−d electronic transition, coming from the electronic state density of this metal (Figure 5b), is centered around 480 nm. It contributes overwhelmingly to the total SFG signal and interferes destructively with the DDT SFG molecular response, giving an SFG spectrum similar to that encountered on a flat gold surface [50]. Nevertheless, it is worth noting that the gold surface density on this nanostructured interface is 4% with respect to the equivalent gold surface area, which strengthens the fact that SFG spectroscopy is sensitive to the chemistry of functionalized nanoparticles. The LSPR effect is hampered by the interband electronic transition in the SFG process because the SFG wavelength coincides with this latter. We will see further in this review that the tuning of the incident laser beam from blue to red allows playing with the gold LSPR and interband electronic properties in order to favor molecular detection. These interference effects in the SFG process can be easily understood through a simple fitting procedure of the SFG intensity which consists in the square norm of the effective non-linear second-order susceptibility $\chi_{\mathrm{interface}}^{\left(2\right)}$ of the interface [53]. Since the optical properties of silicon/glass substrates do not have any significant contribution in the SFG process, they are neglected, which leads to the following expression based on the definitions of mathematical complex values:

$$\begin{array}{cc} {∥\chi_{\mathrm{interface}}^{\left(2\right)}∥}^{2} & ={∥\chi_{\mathrm{substrate}}^{\left(2\right)}+\chi_{\mathrm{adsorbate}}^{\left(2\right)}∥}^{2}={∥\chi_{\mathrm{non}-\mathrm{resonant}}^{\left(2\right)}+\chi_{\mathrm{resonant}}^{\left(2\right)}∥}^{2}\\ & ={∥\chi_{\mathrm{AuNPs}}^{\left(2\right)}+\chi_{\mathrm{molecules}}^{\left(2\right)}∥}^{2}, \end{array}$$

with:

(1) $$\chi_{\mathrm{AuNPs}}^{\left(2\right)}=A\;e^{i\Phi }\;\mathrm{and}\;\chi_{\mathrm{molecules}}^{\left(2\right)}=\sum_{j=1}^{n}\frac{a_{j}\;e^{i\varphi_{j}}}{\omega_{\mathrm{ir}}-\omega_{j}\pm i\Gamma_{j}}.$$

This description is intrinsic to SFG/DFG spectroscopy because of the properties of non-linear second-order susceptibilities (third-rank tensors) [55]. In this equation, the molecular non-linear second-order susceptibility is described as a sum of complex Lorentzian functions (+ for SFG, − for DFG in the imaginary part of Equation (1)) corresponding to each vibration mode (i.e., n=5 in the two previous examples of DODAC and DDT). From these considerations, it is clear that an interference factor (phase shift $\Phi -\varphi_{j}$) between the substrate and the adsorbate will affect more or less strongly the spectral shape as a function of the electronic properties of gold, i.e., its s−d interband transition whether it is nanostructured or not. It is worth noting that the phase shift in the SFG spectra is a unique physical marker of the different components of the interface in the optical range with respect to the information afforded by PM-IRRAS or SERS spectroscopy.

**Figure 6:**
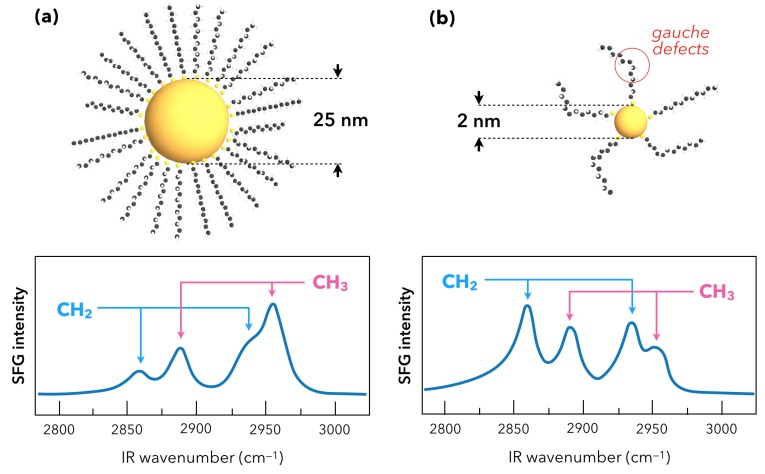
Evolution of the SFG vibrational fingerprint (C-H spectral range) of dodecanethiol molecules (DDT) adsorbed on AuNPs of 2 nm (**a**) and 25 nm (**b**) diameter, respectively. In Figure 6a, the methyl (CH_3_) vibration modes intensity dominates the SFG contribution while in Figure 6b, the methylene (CH_2_) groups dominate the SFG contribution. As detailed in the text and in references from which the picture is adapted [56,57], this is related to the density of gauche defects in the DDT alkane chains depending on the accessible surface and the ability to move in its surroundings.

Afterwards in 2006–2007, Benderskii’s team used SFG spectroscopy as a sensitive probe of the molecular conformation in a nanoscale geometry, which determines the efficiency of the charge (electron) transfer in applications. They used femtosecond laser beam with an incident visible beam fixed at 800 nm wavelength: this enables to get rid of any LSPR or interband contributions likely to interfere with the DDT SFG signal. Moreover, in order to favor the surface reflectivity on their CaF_2_ substrate, they performed SFG spectroscopy in ssp-polarization configurations (*s*: SFG beam, *s*: Visible beam, *p*: IR beam) which is more efficient than in ppp-polarization for any glass substrate (see Figure 2 for the definition of the polarization plane used in SFG experiments). Once again, this is related to the tensor nature of the non-linear second-order susceptibility of the interfaces $\chi_{\mathrm{interface}}^{\left(2\right)}$ which also includes the surface reflectivity in order to quantitatively model the SFG signal intensity as detailed in reference works for the interested reader [14,50]. Benderskii’s team deduced information about the average orientation (tilt angle) of the alkane chains of DDT molecules (1.6 nm length) adsorbed on submonolayer films of metallic nanoparticles (DDT-capped gold and silver nanoparticles). It was performed for different nanoparticles diameters ranging from 2 nm (Figure 6a) to 25 nm (Figure 6b) deposited on CaF_2_ windows [56,57] and the following trend was observed: the relative SFG intensity of the methylene (CH_2_) vibration modes increased with respect to the methyl (CH_3_) ones as the AuNP size decreased (Figure 6). Contrary to what is observed on flat gold surfaces where well-ordered DDT molecules build a stable SAM (all-trans conformation) and where only the three methyl vibration modes were detected [50], the presence of methylene vibrations is a well-known indication of the presence of size-dependent gauche conformational defects. In other words, the smaller the AuNP radius, the bigger the surface curvature, which gives rise to gauche defects in the alkane chain due to the greater surface to volume ratio that can be probed by the DDT molecules during adsorption. A comparison with classical IR absorption and Raman measurements was performed on samples with similar preparation protocols but with thicker layers, given the weaker detection limits of those techniques: no systematic/coherent change was detected in the respective intensities of methyl and methylene vibration modes. This experiments perfectly illustrate the high intrinsic sensitivity of SFG to molecular conformation on flat or curved surfaces. Besides, the same trend was observed for similar experiments performed on silver nanoparticles (AgNPs) [57]. Regarding silver, it should also be noted that a 2C-SFG experiment was conducted in 2008 by Traverse and co-workers on a stack of nine layers of AgNPs (1 nm) embedded in Si_3_N_4_ layers (9 nm thick) on silicon. In this experiment, a weak SFG signal related to the 2D electronic states of this special structure only appeared when the incident visible beam energy matched that of the AgNP SPR located at 420 nm [58].

**Figure 7:**
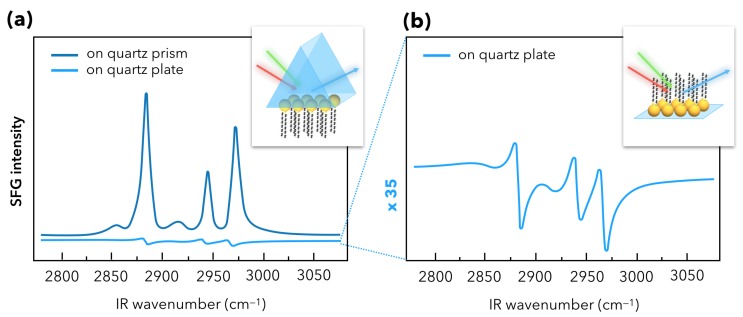
(**a**) SFG spectra of DDT molecules grafted on quartz surface (prism) in a total internal reflection SFG (TIR-SFG) configuration compared with the classical SFG reflection configuration on a quartz surface (plate). (**b**) Zoom of the SFG spectrum of the plate surface, illustrating the great differences in the respective spectral shapes and intensities. It is worth noting that the SFG spectra of the methyl (CH_3_) vibration modes pointing out from the probed surface are strongly enhanced in the TIR-SFG configuration (35 times higher). The latter show that DDT molecules adopt a well-organized conformational order inside the self-assembled monolayer (SAM). Picture adapted from reference [59].

A specific experimental improvement was achieved by Tourillon and co-workers from 2007 to 2009 in order to develop highly sensitive platforms based on AuNPs in an original optical scheme where SFG spectroscopy is performed in total internal reflection configuration for the IR, visible and SFG beams (TIR-SFG) as depicted in Figure 7a [59,60]. It was based on a previous experimental demonstration that the metal [61] or molecular [62] SFG yields can be increased by coupling one of the input waves (IR or/and Visible) to a surface polariton at the metal-air interface [62]. Tourillon’s idea was to take profit of the building of dense AuNP monolayers or close-packed arrays of AuNPs adsorbed on a quartz prism in order to boost the SFG sensitivity. He could enhance the SFG signal of adsorbed dodecanethiol molecules of at least one magnitude order (with respect to a flat quartz plate where the SFG signal is detected in classical reflection, Figure 7b) [59]. He could also monitor the adsorption of avidin in the biotin-avidin recognition process [60], as the biosensor archetype [63]. It is worth noting that in the TIR-SFG configuration, the symmetry breaking of the interface is also coupled to a supplementary breaking of the local electric fields intensities (exponential decay of the electric field amplitude) involved in the non-linear process around the AuNPs, which could favor even more the enhancement of the SFG signal coupled to the LSPR phenomenon. Accordingly, the TIR-SFG configuration is exploited to address issues related to biological materials as developed in the Section 3.1 entitled “Towards applications of gold nanoparticles”.

**Figure 8:**
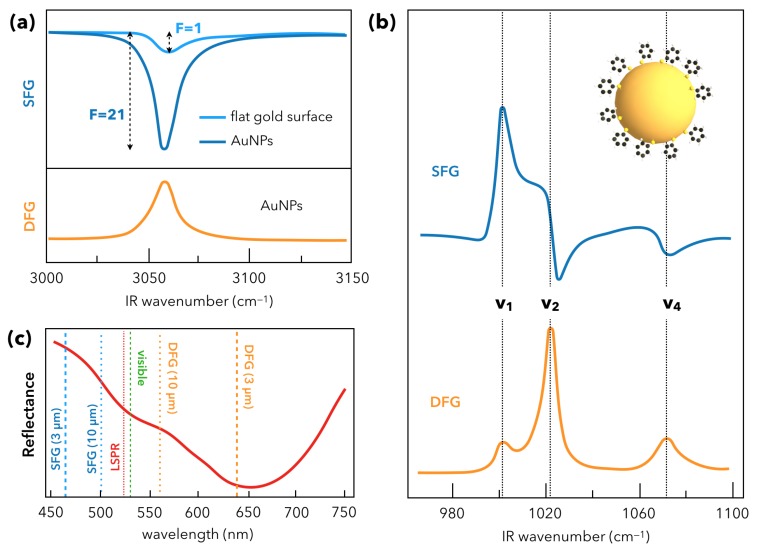
SFG/DFG signals as a function of the UV-Visible optical/electronic properties of nanostructured gold. (**a**) Comparison between thiophenol/Au(111) interface and thiophenol/AuNPs/APTES/Si interface (Visible = 532 nm, IR = 3 µm) in the C–H spectral range (stretching vibration mode of the phenyl ring). In the AuNP case, SFG intensity is enhanced by a factor *F* = 21, corresponding to evidence of LSPR-SFG coupling. The DFG spectrum is also displayed to illustrate the different interference role of the s−d interband electronic transition of gold on the SFG/DFG spectral shapes. (**b**) SFG/DFG spectra of thiophenol/AuNPs/APTES/Si interface (Visible = 532 nm, IR = 10 µm) in the spectral range of C–C vibration modes of the phenyl ring, showing a high SFG and DFG response of this strongly Raman-active modes, also shape-modulated by the interband electronic s−d transition. (**c**) UV-Visible spectrum of the thiophenol/AuNPs/APTES/Si interface. The presence of silicon induces a shape-reversal of the optical response, dominated by the silicon reflectivity. The LSPR maximum is located at 525 nm and couples more or less with the SFG/DFG energy (in addition to the incident visible beam one) as a function of the probed IR spectral range. The LSPR-SFG coupling is favored in the (**b**) case. Figure panel is adapted from references [64,65].

All the above-mentioned works do not try to model quantitatively the would-be coupling between AuNPs LSPR and the SFG response of surrounding molecules. A quantitative approach to this problem of combining SFG spectroscopy and plasmonics in AuNPs was first addressed by Pluchery and co-workers in a series of three publications during the 2009–2013 period [64,65,66]. In these works, the molecular probe was thiophenol (C_6_H_5_SH), a well-known reference molecule acting as a chemical pollutant to be degraded by (photo)catalysis supported by plasmonics. As a consequence, an abundant literature is related to this chemical organic compound which tackles this subject through linear and non-linear optical spectroscopy. Modelling is elaborated through density functional theory calculations of this molecule adsorbed on gold clusters for instance [67]. In a first step, the idea was to find a reference sample with respect to the nanostructured interface depicted in Figure 2 (Thiophenol/AuNPs/Si) to calculate an enhancement factor directly related to the AuNP LSPR (17 nm diameter) [64]. This naturally led to consider a flat crystalline surface of gold Au(111) covered by an organic SAM adsorbed by dipping the metal substrate in a solution of 1 mM thiophenol. By keeping identical conditions for the SFG measurements on the two samples, it has been shown that the SFG intensity enhancement factor *F* of the C–H stretching vibration mode (C–H of the phenyl ring: 3058 cm^−1^) was 21 on the AuNP interface with respect to the Au(111) one, as illustrated in Figure 8a. It is explained by the energy of the incident visible laser beam (at 532 nm) which is located near the LSPR maximum absorbance (520 nm). However, the interband character of gold is always conserved in both samples leading to a dip-shaped spectral feature of the (C–H)-vibration. It is worth noting that the silicon electronic properties in the blue-violet (i.e., matching the SFG beam energy) also contributes to the non-resonant SFG background. We must likewise consider that there is only a filling factor of 10% of gold on the nanostructured sample with respect to Au(111) one when the incident IR and visible beams probe the same area. By comparing the *F* factor with the enhancement factor *T* of the local electric field intensity at the surface of a gold sphere in the electrostatic approximation of the Mie theory, the authors also showed that *T* has a similar value of 20. Besides, a supplementary comparison with the DFG response of the nanostructured sample shows that the dip-shaped feature observed in SFG becomes peak-shaped in DFG because of the sign change in the imaginary part of the Lorentzian function defined in Equation (1). Moreover, as the DFG energy is located at the edge of the interband electronic transition of gold, the non-resonant contribution is weaker than in the SFG case. While this first experimental quantitative illustration of the SFG-LSPR coupling was unambiguous, it was necessary to develop a complete modeling of the SFG intensity of the nanostructured interface related to the fundamentals equations of SFG spectroscopy, especially by calculating the contributions of the surface reflectivity (Figure 8c) to $\chi_{\mathrm{interface}}^{\left(2\right)}$. It has been addressed and generalized in a second step by considering SFG and DFG measurements performed with the CLIO free electron laser (FEL) in Orsay (France) [65], allowing reaching of a different IR spectral range centered around 10 µm (Figure 8b), i.e., in a vibrational range where the strong Raman-active vibration modes of the thiophenol phenyl ring could be probed, again on samples depicted in Figure 2.

Therefore, the authors use the Maxwell-Garnett formalism in order to calculate the effective dielectric constant $\epsilon^{*}={\left(n^{*}\right)}^{2}$ of the AuNPs by considering they are embedded in a host air matrix. In this way, it is possible to calculate the weight of the surface reflectivity in the non-linear second-order susceptibility. The knowledge of this optical parameter is crucial because it fixes or not the existence of the LSPR-SFG coupling, beyond a simple absorbance/reflectance effect of the surface. Indeed, it is evidenced that the normal contribution of this reflectivity (i.e., in the *z*-direction with respect to the sample surface plane) is not sufficient to explain the differences observed in the SFG and DFG spectra intensities of the phenyl ring vibration modes: these measurements and modeling proved that the existence of the coupling of SFG with plasmonics was consistent between the nanoscale (AuNPs) and the microscale (i.e., whatever the description used for the surface area probed by the incident IR and visible laser beams). Besides, at the molecular scale, it was shown that SFG/DFG measurements were consistent with DFT calculations [67] performed on a thiophenol molecule adsorbed on a gold atom, allowing deduction of the nature of the three vibration modes which was a long haul task especially for the 1075 cm^−1^ (*v*_4_ in Figure 8b). The latter appears at this position after undergoing an energy blueshift of 15 cm^−1^ because of the thiophenol adsorption on gold. In summary, it is clear that the energies of the Visible/SFG/DFG processes of the interface of Figure 3 can match more or less efficiently the LSPR energy and favor or not the LSPR-SFG/DFG coupling. The spectral shape of the UV-Visible reflectance conditioned by the silicon reflectivity and the AuNP aggregates as observed in Figure 8c shows that it is possible to tune the incident IR and visible laser beams to fix that point. The best experimental way to answer this question would be to perform 2C-SFG spectroscopy by tuning the incident visible laser beam from blue to red in order to monitor and quantify the LSPR-SFG coupling. This point will be discussed in the section entitled “Recent developments in SFG spectroscopy of plasmonic materials”.

Finally, the monitoring of the AuNPs aggregates depends on their surface coverage on silicon and strongly influences the SFG/DFG spectral shapes as it is discussed exhaustively in the last reference [66] in the series. Considering four different AuNP surface coverages ranging from 1% to 15%, it has been shown that according to the formalism of the effective medium (Maxwell-Garnett or Bruggeman) chosen in the framework of the three-layers model, the deviations to the expected linear law between the vibration mode amplitude (thiophenol C–H streching vibration) and the surface coverage rate by AuNPs could only be explained by the presence of the LSPR. It is worth noting that this linear law is only valid for SFG/DFG if the phase-matching condition between the three beams involved in the non-linear process is satisfied, which is the case in the actual configuration. Otherwise, in the case of dispersed nanoparticles (matrix, solutions), the non-linear phenomena can be scattered in all the directions with respect to the reflected SFG beam coming from a flat surface. In these conditions, the above-mentioned linear relation is not valid [68,69].

## 3. Recent Developments in SFG Spectroscopy of Plasmonic Materials

### 3.1. Towards Applications of Gold Nanoparticles

**Figure 9:**
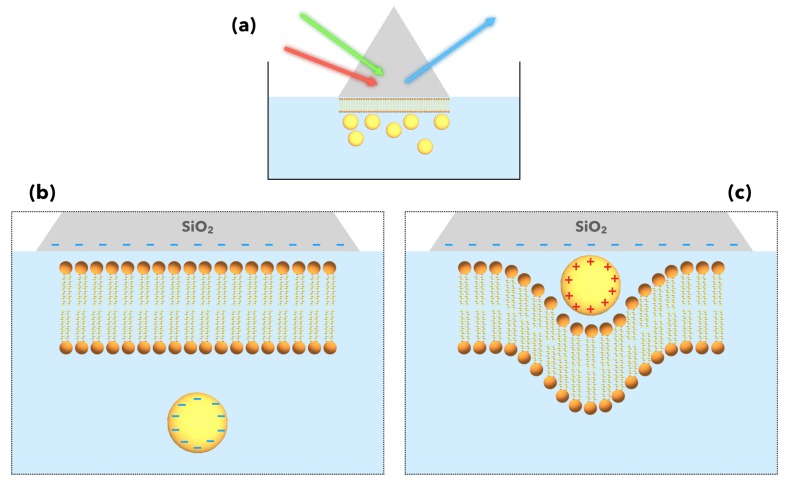
(**a**) Applications of SFG spectroscopy to the study of the interactions of AuNPs with biological material in aqueous medium. (**b**) Effect of a total negative surface charge (SiO_2_ and AuNPs) on the nanoparticle interaction with a model membrane (lipid bilayer) where no penetration is observed. (**c**) same as (**b**) but for positively charged AuNP surface. This time, the AuNPs crosses and distorts the lipid bilayer to reach the SiO_2_ surface. Figure panel is reprinted (adapted) with permission from [74], copyright 2016 American Chemical Society.

From that moment, thanks to all these fundamental results, it is possible to apply SFG spectroscopy to the study of more complex interfaces related to practical issues. For instance, the increasing role of nanoparticles in consumer goods or medical applications raises questions regarding their possible toxicity for humans. Indeed, as AuNPs are engineered for targeted drug delivery, it is mandatory to understand their physico-chemical interactions with their biological environment, especially in the case of cell damage. SFG spectroscopy/microscopy has already proven to be of great interest in the study of model biointerfaces in the past [70,71]. In fact, the studies of interactions of magnetic nanoparticles [72] or AuNPs [73] with cell membrane models are performed on more or less complex interfaces made of supported lipid bilayers. Indeed, small AuNPs may interact with the cell membrane but the interaction mechanism at the nanoscale is not well understood. To solve these issues, Cecchet’s group examined in 2016 how the organization of supported lipid membranes may be affected by the surface charge of small AuNPs (5 nm diameter) and the substrate on which they are adsorbed, all in an aqueous medium [74] as illustrated in Figure 9a. By charging the AuNP surface negatively (Figure 9b) or positively (Figure 9c) depending on their ligands, they showed how the AuNPs induce (or not) structural damages to DPPC (1,2 Dipalmitoyl-*sn*-glycero-3-phosphocholine) membrane models deposited on SiO_2_ (negative surface charge) and CaF_2_ (positive surface charge) by performing SFG measurements before and after injection of the AuNPs in the water as a function of the exposure time to these latter. It is achieved in the C–H and O–H spectral range to probe the conformational order of the lipid structure and the water molecules in its hydration layer. They observe that all the critical structural changes occur in less than 90 min whether the AuNPs interact (Figure 9c) or not (Figure 9b) with the DPPC bilayer. Moreover, whereas it is not explicitly mentioned in all these works, the LSPR-SFG coupling should also act preponderantly in the high SFG intensity of the vibration modes especially since it is measured in the TIR-SFG configuration with the incident visible beam with its energy matching the SPR one developed by Tourillon as mentioned above [59,60]. They also take advantage of the TIR-SFG configuration in a recent work [75] to extend their investigation to the interaction of cationic AuNPs with supported model membranes carrying negative charges in order to mimic living systems where the outer and inner membrane sides of the cell are negatively charged.

**Figure 10:**
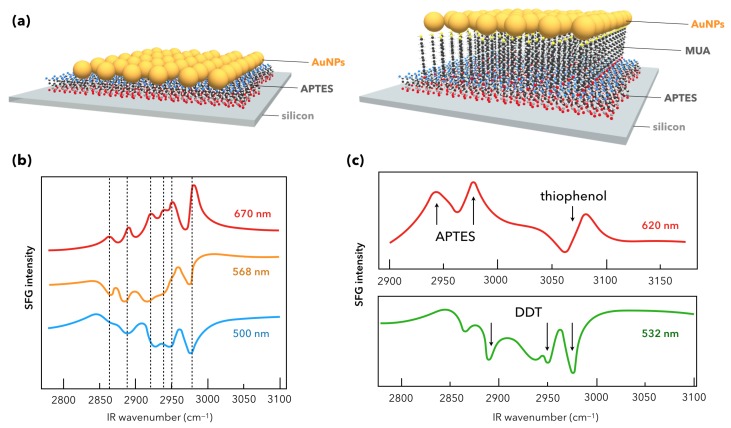
(**a**) AuNPs/APTES/Si (or AuNPs/MUA/APTES/Si) interfaces before functionalization with thiophenol or dodecanethiol (DDT). (**b**) 2C-SFG spectra of the AuNPs/APTES/Si interfaces for three different incident visible wavelengths from blue to red as indicated on the picture. The complete shape-reversal of the APTES vibrational signature between the blue and red curves is well marked. (**c**) SFG spectra for specific incident wavelength revealing the vibrational fingerprint of thiophenol (**top**) or DDT molecules (**bottom**) chemically adsorbed on two different AuNPs/APTES/Si interfaces, respectively. Figure panel is adapted from reference [34].

To date, in the research field of AuNPs potentially used as highly sensitive platforms in (bio)sensing for (bio)molecular recognition, there is only one work published in 2015 [34] which treated quantitatively the subject after the emerging work of Tourillon in 2009 [60], but not in TIR-SFG conditions. Contrary to this latter, Dalstein and co-workers choose silicon as a substrate to build a DDT (or thiophenol)/AuNPs/Si interface similar to Figure 2. For such a platform, the idea is to use afterwards silicon as an electronic transducer of the optical SFG response, which requires a good understanding of the optical property’s role in the SFG response of the building blocks. In this paper, the focus is put on the deep investigation of the grafting APTES or APTES/MUA (MUA stands for mercaptoundecanoic acid) layer on silicon before and after AuNPs (15 nm diameter) immobilization thanks to this organic layer. The substrate is thus silanized with either amine or mixed aminethiol layer as depicted in Figure 10a. The effect of these small AuNPs on the SFG signal of the grafting layer (6 methyl/methylene stretching vibration modes) is probed by 2C-SFG spectroscopy at different incident visible wavelengths (500 nm, 568 nm, 670 nm) showing a shape-reversal from dips to peaks when tuning the color from blue to red as displayed in Figure 10b. Once again, this interference effect comes from the non-resonant SFG signal of the AuNP interband electronic transition and the silicon optical activity but, this time, it is directly highlighted by tuning the incident visible beam wavelength in the SFG process and not by indirect deduction coming from the SFG and DFG shapes of the vibration modes as evidenced in Figure 8a,c. Thanks to the optical properties of gold, 2C-SFG enables a clear localization of the six weak SFG vibration modes of the disordered APTES layer without taking profit of a TIR-SFG configuration (which is not possible with silicon in the probed optical spectral range).

Despite the presence of APTES or APTES/MUA, it is shown afterwards that the AuNP functionalization by DDT (Vis = 532 nm, Figure 10c) or thiophenol (Vis = 620 nm, Figure 10c) is successful and can be monitored by 2C-SFG spectroscopy even in the presence of a grafting layer. Besides, this works enables to establish a linear relationship between the AuNP absorbance with respect to their surface coverage in UV-Visible spectroscopy, which proves valid up to the aggregation limit: this gives an easy tool to evaluate the AuNP surface coverage of any similar sample from UV-Visible spectroscopy without systematically resorting to Scanning Electron Microscopy (SEM). Moreover, this work shows that the SFG intensities follow a quadratic relation with the UV-Vis absorbance, a direct proof that there exists a close correlation between linear and non-linear optical properties. From the previous work, it is clear that the LSPR-SFG coupling in such interfaces can be probed by 2C-SFG spectroscopy on a wide spectral range of incident visible wavelengths.

### 3.2. Complex Plasmonic Nanostructures

**Figure 11:**
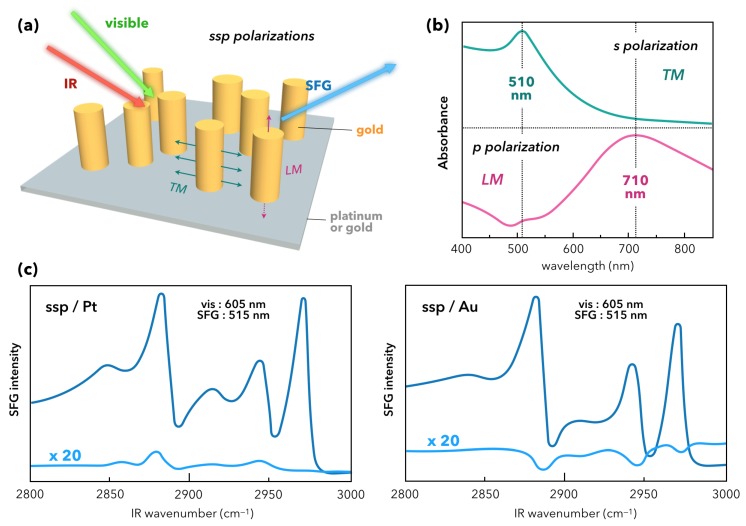
(**a**) Sketch of the 2C-SFG experiment on the gold nanopillars electrochemically grown on a gold or platinum surface in ssp-polarization scheme. Samples are functionalized with dodecanethiol (DDT) molecules. The electric yield enhancement of the transverse (TM) and longitudinal (LM) surface plasmon modes is depicted in green and red, respectively. (**b**) UV-Visible absorbance spectra of the sample with a gold substrate. (**c**) Comparison between the SFG spectra of the DDT/Au nanopillars/Pt (**left**) or Au (**right**) interfaces (dark blue) with respect to the DDT/Pt (**left**) or Au (**right**) interfaces (light blue). Figure panel is adapted from reference [77].

In the previous sections, we have seen that the emphasis has been put on small spherical nanoparticles of gold covered by molecular probes or in interaction with biomaterials. We also observed that different strategies were developed to take advantage of the AuNP LSPR in order to enhance the chemical response in non-linear SFG spectroscopy. While the use of spherical AuNPs is interesting when trying to apply SFG spectroscopy to (bio)chemical or biological applications, the plasmonic contribution is not systematically optimized in such systems. Indeed, as SFG spectroscopy is intrinsically sensitive to the symmetry breaking of the electronic properties of the studied interface, it becomes obvious that spheres (centrosymmetrical materials) are not the best candidate to take full advantage of plasmonics. The point is that as spheres are adsorbed on an interface, the SFG signal appears because of the symmetry breaking related to the different chemistry above and below the AuNPs. In plasmonics, materials can be designed to optimize the SERS signal of highly nanostructured surfaces [76]. This begins to be equally applied in non-linear optics for nanopillars and nanotriangles. In fact, the first 2C-SFG measurements on highly nanostructured platforms were performed on gold nanopillars vertically standing on metallic surfaces (gold or platinum) by Cecchet’s group [77]. As displayed in Figure 11a, the nanopillars are electrochemically grown on the substrate with an average height of 106 nm or 100 nm and a mean diameter of 66 nm or 80 nm in the case of gold and platinum, respectively. Within this framework, both samples are functionalized by DDT molecules. Gold nanopillars exhibit two LSPR: a transverse mode (TM) located at 510 nm and a longitudinal mode (LM) located at 710 nm or 660 nm depending on the growth on gold or platinum, respectively. The highlighting of these modes by UV-Visible spectroscopy requires the monitoring of the incident beam polarization (*s* for TM and *p* for LM) as depicted in Figure 11b. In these conditions, it is clear that playing with the polarization scheme in the 2C-SFG process along with the incident visible laser beam wavelength (between 450 and 670 nm) allows fine tuning of the enhancement of each LSPR and therefore optimize the LSPR-SFG coupling as demonstrated in Figure 11c by the enhancement of the molecular SFG signal of the DDT adsorbed on the samples. From these observations, it is shown that the coupling is the most efficient for a ssp-polarization scheme with a visible or SFG wavelength in coincidence with the TM LSPR, i.e., 515 nm. It is worth noting that this value of 515 nm is obtained for the SFG wavelength when the incident visible beam is set to 605 nm because the IR probed spectral is centered around 3.45 µm. With respect to the equivalent flat gold or platinum surface covered with DDT, the enhancement factor of the methyl stretching vibration modes in SFG reaches 20 thanks to the nanopillars. A similar (but weaker) behavior is observed when exciting the LM LSPR in ppp-polarization scheme but for an incident visible wavelength matching its maximum absorbance at 650 nm. An extended discussion in this paper allows determination that the DDT contribution to the SFG signal mainly comes from the nanopillars due to the local electric field enhancement by the TM and LM LSPR along the lateral surface and the top of the nanopillars, respectively. It is worth noting that the s−d interband electronic transition of gold always interferes with the molecular SFG signal whether there exists an LSPR-SFG coupling or not. Anyway, this work proves that 2C-SFG spectroscopy can probe spatially the chemistry of plasmonic platforms and give complementary information to SERS spectroscopy.

**Figure 12:**
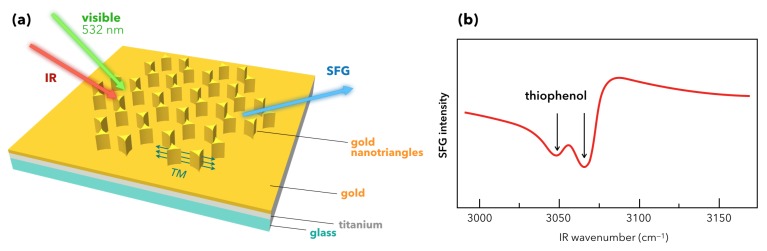
(**a**) Sketch of the SFG experiment performed on gold nanotriangles (AuNT) grafted by nanosphere lithography (NSL) on a gold film (30 nm thickness) deposited on a titanium layer (2 nm thickness) grafted on glass. The AuNT are structured in honeycombs. The AuNT transverse mode (TM) is equally displayed in green. The interface is chemically functionalized by thiophenol molecules. (**b**) SFG spectrum of the sample of (**a**) in ssp-polarization, revealing the two C–H (phenyl ring) of the thiophenol. Figure panel is adapted from reference [78].

To go further, Barbillon and co-workers make the choice in 2018 to use highly ordered nanostructures made of gold nanotriangles (AuNT, height = 60 nm, lateral side = 150 nm) prepared by nanosphere lithography (NSL) technique. The sample consists in a glass substrate covered by a Ti adhesion layer (2 nm) on which a gold film (30 nm) is evaporated as displayed in Figure 12a [78]. The molecular probe is thiophenol, chemically adsorbed on the plasmonic platform. In SERS spectroscopy, its vibrational fingerprint is easily observed in the spectral range of the phenyl ring (10 µm) [19] but remains silent in the C–H spectral range (3 µm). However, it is possible to detect the C–H vibrations modes by SFG spectroscopy, although the highly nanostructured interfaces obtained by NSL do not play in favor of SFG spectroscopy because of its high centrosymmetric character (*p6mm* class symmetry, structured in honeycombs), contrary to the above-mentioned work where nanopillars are randomly dispersed [77].

Nevertheless, as was observed for highly centrosymmetric AuNPs, the authors evidence by SFG in Figure 12b a localized detection of the thiophenol preferentially on the AuNT. It shows up two weak thiophenol ϵ and σ vibration modes [67] at 3050 cm^−1^ and 3071 cm^−1^ (ssp-polarization scheme, incident visible wavelength at 532 nm), instead of only one as usually seen [64]. The explanation of the enhancement effect and the LSPR-SFG coupling on AuNT is similar to that encountered for gold nanopillars as explained above because of the TM LSPR mode of AuNT (540 nm). The highlighting of these two vibration modes on a surface is made possible by SFG rather than SERS despite a weak filling factor of AuNT (8%). SERS is mainly useful in solution in order to discriminate between ϵ and σ. The issue is to explain why it works better in SFG than in SERS. It comes from the weak/null enhancement factor (EF) for SERS in the C–H spectral range because of the great spectral redshift (120 nm) between the Raman wavelength (545 nm) and the excitation wavelength (665 nm). In other words, in SERS: $\lambda_{vis}=\lambda_{exc}\neq \lambda_{plasmon}$ with $\lambda_{exc}<<<\lambda_{Raman}$. Conversely, in SFG: $\lambda_{vis}=\lambda_{exc}\approx \lambda_{plasmon}$ (532 nm) with a smaller spectral redshift (70 nm) with respect to $\lambda_{SFG}$ (460 nm). These simple relations perfectly illustrate the advantages and complementarities of both SERS and SFG spectroscopies. In summary, over the C–H spectral range, (i) for SERS [19]: no infrared excitation in Raman process; very high sensitivity (vs SFG), depends on gold underlayer thickness; no localized detection of molecules in classical configuration; (ii) for SFG: sensitivity depends on surface geometry and is limited in vertical direction due to strong gold s−d interband electronic transitions; infrared and Raman excitations in SFG process; localized detection of molecules in classical configuration. Finally, this work paves the way towards a direct comparison of SERS and SFG vibrational spectroscopies on complex nanostructured metal interfaces, even with a high degree of centrosymmetry, thanks to the presence of distinct LSPR favoring preferential directions as integrated by the non-linear optical response of the interface.

## 4. Conclusions

Presently, SFG spectroscopy is mostly developed on themes related to chemical and biological issues as for recognition processes leading to a monitored drug delivery for medical issues in the latter case. SFG spectroscopy has become a common optical investigation tool in many research laboratories throughout the world. Restricted to physicists in its early days in the late 1980s, this physical non-linear optical probe of any kind of interface, whatever the nature of its building blocks or its physical state (solid, liquid, gas), has rapidly managed to quickly expand its circle of applications thanks to the involvement of the chemist community. Moreover, it is now possible to find easily commercial femtosecond SFG setups, allowing measurement of dynamic reactions at interfaces immersed or in contact with an aqueous medium. This is also why SFG studies on modified water surface constitute an abundant literature on the subject alongside with specific issues related to biology: protein folding near model cell membranes, flip-flop processes in lipid (hybrid or not) bilayers, as summarized in some references of this review [70,71]. While the study of nanomaterials by SFG has begun 18 years ago, it is today circumscribed to the investigation of interactions in chemical and biological applications. By taking advantage of the LSPR excitation of gold nanoparticles in the SFG process, this latter has the potential to heat the probed interface to facilitate chemical reactions. Optically, when the LSPR energy matches the visible or SFG energy in non-linear optics, it significantly improves the molecular detection threshold. In this review, we have seen that small gold nanoparticles have constituted the core of such SFG studies of nanomaterials so far. It comes from, on the one hand, the presumed nontoxic character of gold for biological applications due to its huge physical and chemical stability and, on the other hand, the fact that its main electronic and optical properties are located in the visible spectral range. The role of its plasmonic and interband electronic properties at nanoscale was the main research field until recently by SFG spectroscopy (picosecond and femtosecond scales) since it is complementary to SERS and PM-IRRAS measurements as non-linear optics provides a different kind of information. Other plasmonic spherical nanoparticles are little or not addressed by SFG due to their lack of chemical stability in air (silver) and their poorer plasmonic properties (copper). The development of 2C-SFG spectroscopy should overcome this limitation within the next years, as it has already been demonstrated on gold for years. Moreover, the use of semiconductor nanoparticles that can be doped and gather free electrons, as those encountered at the surface of metallic nanoparticles, could be relevant if they are combined together in order to manufacture more efficient chemical or biological sensors. Finally, the last part of this review highlights the fact that complementary studies by SERS and SFG spectroscopy of complex nanostructures (nanopillars, nanotriangles) grafted to a substrate open the door to localized non-linear plasmonics. Besides, as the SFG photons are emitted in the visible spectral range, a considerable technical advantage with respect to IR microscopy, it is just a question of time before SFG imaging of nanostructured interfaces be revealed in a close future.
